# Imerslund-Gräsbeck syndrome (selective vitamin B_12 _malabsorption with proteinuria)

**DOI:** 10.1186/1750-1172-1-17

**Published:** 2006-05-19

**Authors:** Ralph Gräsbeck

**Affiliations:** 1 Biochemistry Unit, Minerva Foundation Institute for Medical Research, Biomedicum Helsinki, FI-00290 Helsinki, Finland

## Abstract

Imerslund-Gräsbeck syndrome (IGS) or selective vitamin B_12 _(cobalamin) malabsorption with proteinuria is a rare autosomal recessive disorder characterized by vitamin B_12 _deficiency commonly resulting in megaloblastic anemia, which is responsive to parenteral vitamin B_12 _therapy and appears in childhood. Other manifestations include failure to thrive and grow, infections and neurological damage. Mild proteinuria (with no signs of kidney disease) is present in about half of the patients. Anatomical anomalies in the urinary tract were observed in some Norwegian patients. Vitamin B_12 _absorption tests show low absorption, not corrected by administration of intrinsic factor. The symptoms appear from 4 months (not immediately after birth as in transcobalamin deficiency) up to several years after birth. The syndrome was first described in Finland and Norway where the prevalence is about 1:200,000. The cause is a defect in the receptor of the vitamin B_12_-intrinsic factor complex of the ileal enterocyte. In most cases, the molecular basis of the selective malabsorption and proteinuria involves a mutation in one of two genes, cubilin (*CUBN*) on chromosome 10 or amnionless (*AMN*) on chromosome 14. Both proteins are components of the intestinal receptor for the vitamin B_12_-intrinsic factor complex and the receptor mediating the tubular reabsorption of protein from the primary urine. Management includes life-long vitamin B_12 _injections, and with this regimen, the patients stay healthy for decades. However, the proteinuria persists. In diagnosing this disease, it is important to be aware that cobalamin deficiency affects enterocyte function; therefore, all tests suggesting general and cobalamin malabsorption should be repeated after abolishment of the deficiency.

## Disease name/synonyms

Enterocyte cobalamin malabsorption – Enterocyte intrinsic factor receptor, defect of - Gräsbeck-Imerslund disease – Imerslund-Gräsbeck syndrome (or disease) – Megaloblastic anemia 1 – Selective vitamin B_12 _malabsorption with proteinuria.

Nowadays the most frequently used name is Imerslund-Gräsbeck syndrome, abbreviated to IGS.

## Definition

The Imerslund-Gräsbeck syndrome (IGS) is a rare autosomal recessive disorder characterized by vitamin B_12 _(cobalamin) deficiency due to selective malabsorption of this vitamin and usually resulting in megaloblastic anemia appearing in childhood (but not immediately after birth) and which is responsive to parenteral vitamin B_12 _therapy. Mild proteinuria is frequently but not always present. Neurological symptoms typical of cobalamin deficiency have been reported [1] but are seldom pronounced.

## Epidemiology

To date, 27 patients from 19 families in Finland (population 5.2 million) and 19 from 15 families in Norway (population 4.6 million) have been reported (unpublished data and Dr. H. Broch, personal communication from). This gives an estimated prevalence of <6:1,000,000. About 300 cases have been published worldwide, with new cases predominantly appearing in eastern Mediterranean countries. However, many cases may be misdiagnosed.

## Clinical description

Patient age at diagnosis varies from a few months to about fourteen years old. Patients usually present with non-specific health problems such as failure to grow and thrive, recurrent gastrointestinal or respiratory infections, pallor and fatigue. Detailed examination reveals anemia and/or proteinuria. Typical features of megaloblastic anemia may be observed together with neurological signs, which have been reported as mild in the published cases. Close relatives may show similar clinical manifestations.

Megaloblastic anemia is caused by inability of the hematopoietic cells to divide, due to failure of DNA replication. All dividing cells are affected, especially rapidly replicating ones, such as mucosal and sperm cells [2]. In principle, anemia is not an obligatory sign of cobalamin deficiency, which may manifest itself as a purely neurological disease and possibly in other ways (protracted infections, amblyopia, *etc*.). Also, there is increasing evidence that subclinical deficiency of cobalamin contributes to the development of atherosclerosis, dementia and osteoporosis especially in the aged patients [3-5]. However, according to the author's experience, most cases of IGS present with typical megaloblastic anemia, perhaps with the exception of patients detected by examining the relatives of diagnosed cases.

Proteinuria often, but not always, accompanies IGS. A study of 13 Finnish patients found that moderate or clearly evident proteinuria was present in six of the subjects. The proteinuria was neither typically glomerular nor tubular [6]. Similar findings were obtained in a Norwegian study [7]. Both studies indicated that in properly treated patients, the proteinuria persisted over the years without noticeable change and that kidney function did not deteriorate. In some of the original Norwegian cases, congenital abnormalities of the urinary tract (double ureters, horseshoe kidney) were found [8], but in recently diagnosed cases such anomalies have been absent (H. Brock, personal communication).

Association of IGS with a number of congenital diseases and anomalies has been reported (beta-thalassemia, dolicocephaly, chromosomal deletion) but they appear not to be linked to this condition [9].

## Etiology

### Physiology of vitamin B12 transport

The intestinal absorption of cobalamin involves a number of steps and a disturbance in any of them may cause cobalamin malabsorption resulting in cobalamin deficiency. Under physiological conditions, the vitamin is liberated from food by digestive enzymes and subsequently becomes bound to the glycoprotein haptocorrin (also called R-protein or cobalophilin), which is present in leukocytes, saliva and other secretions. In the small intestine, the change in pH and action of pancreatic enzymes result in vitamin dissociation from haptocorrin. The vitamin is then available for binding to the intrinsic factor secreted by the gastric mucosa. The resulting cobalamin-intrinsic factor complex attaches to a receptor in the distal small intestine (ileum) in the presence of calcium ions. Subsequently, the complex is internalized, the vitamin liberated and transferred to transcobalamin (also called transcobalamin II) synthesized in the intestine (probably in the vascular endothelium) and elsewhere. From the blood the transcobalamin-cobalamin complex delivers the vitamin to the tissues by attaching to specific receptors. Lack of transcobalamin results in rapid development of megaloblastic anemia after birth and also failure to absorb cobalamin from the intestine [10,11]. Some steps in the transport of the vitamin across the intestine may still not be understood [12].

### Pathophysiology of vitamin B12 transport and genetics

IGS is caused by a selective incapacity to transport vitamin B_12 _across the intestinal wall, and is not related to a lack of gastric intrinsic factor. Theoretically, a similar condition could be caused by malfunction of any component in the process transferring the vitamin bound to intrinsic factor in the lumen of the distal small intestine to the specific cobalamin carrier in the blood, transcobalamin. The name "Selective vitamin B_12 _malabsorption with proteinuria" was used to describe the condition when it was reported in Finland in 1960 [13]. A very similar condition was described simultaneously in Norway by Olga Imerslund [8] under the name "Idiopathic chronic megaloblastic anemia in children". Five of the 10 Norwegian cases had congenital abnormalities of the urinary tract. Absorption tests with radioactive vitamin B_12 _later demonstrated that the basic error in these cases was also a specific cobalamin absorption defect [14]. However, a recent study of both populations [15] identified mutations in two different genes: the cubilin (*CUBN*) gene in the Finnish families (three mutations) and the amnionless (*AMN*) gene in the Norwegian families (two mutations). In Turkey, Israel and Saudi Arabia two different *AMN *mutations and three different *CUBN *mutations have been found [15]. It was concluded that the Scandinavian cases result from enrichment of founder mutations, whereas in the Mediterranean region the high degree of consanguinity leads to offspring with rare mutations in both genes.

*CUBN *and *AMN *encode the two subunits (cubilin and amnionless) of the cobalamin-intrinsic factor receptor of the ileal mucosa [15]. The *CUBN *gene is located on chromosome 10 and *AMN *on chromosome 14 [16]. The gene map loci are 10p12.1 [17,18] and 14q32 [16], respectively. The cubilin-amnionless complex is called *cubam *and is considered to be essential for intestinal cobalamin uptake, renal protein reabsorption and early rodent embryogenesis. Amnionless is thought to bind to the amino-terminal region of cubilin and to direct the subcellular localization and endocytosis of cubilin with its ligand. Biallelic mutations affecting either of the two proteins result in IGS in men and dogs [19].

Recent studies aimed at identifying the causative genes and molecules in suspected cases of IGS, revealed defects in the gastric intrinsic factor gene (*GIF*). Thus, these cases cannot be classified as IGS [20] and correspond to other vitamin B_12 _deficiency syndromes, notably pernicious anemia. Evidently, many of the published cases of IGS may not be correctly diagnosed.

### Phenotype-genotype correlations

Congenital anomalies were rare in the Finnish patients carrying the *CUBN *mutation but urinary tract anomalies were observed in the Norwegian patients carrying the *AMN *mutation. This is not surprising given that studies in mice have shown that amnionless is essential for gastrulation [21].

### Environmental factors

In 1995 it was noted that after the initial reports, few new cases of IGS appeared. One explanation for this could be a change in environmental factors, such as the diet [17]. However, during the last decade the author has seen new Finnish cases appear at approximately the rate expected from the prevalence estimate (see the Epidemiology section).

### Animal model

IGS has been found in dogs, first in Giant Schnautzers [22]. He *et al. *[23] demonstrated a linkage of canine IGS to chromosome 8 in a region orthologous to human chromosome 14q32.2-ter, in an interval that contains the *AMN *gene. Further studies showed that effective cubam expression does not occur in the absence of amnionless. Cubilin and amnionless thus belong to the group of membrane receptors that rely on the accessory activity of a separate gene product for trafficking and membrane expression [24].

## Diagnosis and differential diagnosis

In practice, the clinical starting point is detection of macrocytic and/or megaloblastic anemia and possibly proteinuria when a child in a generally poor condition is subjected to laboratory tests. A number of pathogenetic factors and mechanisms may cause the anemia, but the predominant causes are deficiencies of folate and cobalamin. Lack of folate is common in developing countries but rare in the Western world, except among socio-economically disadvantaged groups, in non-breast-fed or chronically ill children and in those suffering from celiac disease. Cobalamin deficiency usually causes some neurological or psychiatric symptoms. A number of laboratory tests are helpful in diagnosing and differentiating between folate and vitamin B_12 _deficiency. They will be discussed in the next section, however, none of them can be regarded as the "gold standard". Diagnosis *ex juvantibus *may be reached by observing a clear-cut response to treatment with small oral doses of folate (the dose should not exceed a few micrograms as larger doses may cause neurological damage in patients with cobalamin deficiency) or vitamin B_12 _injection. When the presence of vitamin B_12 _deficiency has been established, the next step is to determine its cause.

There are several major causes of vitamin B_12 _deficiency. Exposure to nitrous oxide causes cobalamin deficiency by destroying the cobalamin coenzymes. As plants are lacking in vitamin B_12_, a totally vegetarian diet may cause cobalamin deficiency, but because of high folate intake, the signs tend to be more neurological than hematological. Cobalamin deficiency has consequently been found in babies of nursing mothers that abuse laughing gas (nitrous oxide) or are Vegans. In some countries, these may be the most common causes of cobalamin deficiency in babies. A usual cause of cobalamin deficiency is poor absorption of vitamin B_12_. Consequently, gastrointestinal morphology and function need to be examined, including the use of tests for general and cobalamin absorption. Relatively specific cobalamin malabsorption may occur in some cases of general malabsorption (celiac disease or tropical sprue) or due to a pathological intestinal flora in connection with intestinal blind-loops or diverticulosis. Fish tapeworm infection is known to cause cobalamin deficiency in children. Though rare, genuine pernicious anemia also occurs in children and it is caused by intrinsic factor deficiency due to atrophic gastritis. Intrinsic factor may also be congenitally lacking or misassembled. A recent study reported that IGS is difficult to distinguish from cases caused by mutations in the *GIF *(gastric intrinsic factor) gene [20]; however, these difficulties can probably be explained by radiocobalamin absorption tests having been performed during the deficiency state or not at all (see below). Transcobalamin deficiency and errors in the biosynthesis of cobalamin coenzymes result in severe illness shortly after birth.

### Summary of diagnostic steps

In conclusion, the diagnosis of IGS is reached by first detecting the presence of cobalamin deficiency, then demonstrating that this vitamin is poorly absorbed, then excluding other known causes of vitamin B_12 _malabsorption and finally, by showing that after correction of the deficiency state, the only nutrient to be poorly absorbed is vitamin B_12_. Benign and therapy-resistant proteinuria is a helpful, but not an obligatory sign of IGS. Another approach is mutational analysis of the genes concerned (see below).

## Diagnostic methods

Macrocytic and megaloblastic anemias are diagnosed by analysis of peripheral blood and bone marrow, which reveals low hemoglobin, pancytopenia, increased red cell size objectively indicated by high mean corpuscular hemoglobin (MCH) and mean corpuscular volume (MCV) indexes, and typical morphological changes in both red and white cells and platelets and their precursors, *e.g. *megaloblasts, granulocytes with polylobulated nuclei, *etc. *[25]. The myelogram helps to rule out malignant and other conditions that may resemble megaloblastic anemia.

As stated, the main causes of megaloblastic anemia are lack of folate or cobalamin. Rare causes include congenital disturbances in nucleic acid metabolism (*e.g. *orotic aciduria) and lack of other B vitamins. Assay of total serum cobalamin and folate often differentiates between the two deficiencies. Also, a therapeutic test with cobalamin or a few micrograms of folate is helpful. A clear-cut positive response (*i.e. *reticulocytosis, rise in the concentration of blood cells and improved neurological and general condition) is diagnostic.

Although numerous more advanced tests for diagnosis of vitamin B_12 _deficiency exist [26,27], there is no consensus as to which one is the best. Commonly used tests are determination of the serum total vitamin B_12 _or transcobalamin-bound vitamin (holo-transcobalamin) concentrations. Measurements of the metabolites methylmalonate and homocysteine are also used. In principle, methylmalonate accumulates in cobalamin deficiency but not in lack of folate.

The next step is to reveal the cause of the deficiency, which, as described above, may be low intake, poor absorption, destruction of the coenzyme forms by nitrous oxide, transcobalamin deficiency, *etc. *The predominant cause of cobalamin deficiency is poor absorption. This is the case in genuine pernicious anemia, which also occurs in children. The patients do not secrete gastric intrinsic factor because of atrophic gastritis, which is accompanied by antibodies against parietal cells and gastric intrinsic factor; presence of the latter antibodies is highly suggestive of pernicious anemia. Intrinsic factor may also be measured in gastric juice and found to be lacking, sometimes congenitally. In other kinds of cobalamin malabsorption (including IGS) it is present.

Until recently, absorption tests with radiocobalt-labeled cobalamin were routinely used to study patients with megaloblastic anemia and related conditions. Schilling's urinary excretion technique was the most popular method [28]. A small standard dose (usually 1 **μ**g) of radiocobalamin is first given by mouth. This is followed by a parenteral injection of a large dose (usually 1 mg) of non-radioactive cobalamin. Most of this "flushing dose" cannot be retained and is excreted in the urine together with the radioactivity, the amount of which reflects the quantity of radiocobalamin absorbed by the intestine. If the urinary radioactivity is low, cobalamin is probably poorly absorbed. In this case, the test is usually repeated by giving intrinsic factor with the test dose. If this results in increased absorption (and excretion) of radioactivity, lack of intrinsic factor is the cause of the cobalamin malabsorption, *i.e. *the patient has pernicious anemia [28]. If the excretion of radioactivity does not increase, this is called a "malabsorption response". To elucidate the cause of this response, the test may be repeated after treatment (*e.g. *expulsion of tapeworm) or by giving various substances (*e.g. *food, enzymes, antibiotics) orally together with the radioactivity. As detailed in the original article describing IGS [13], these tests allow the known causes of vitamin B_12 _malabsorption to be systematically eliminated. Intrinsic factor activity may also be measured in this way or by immunoassay of gastric juice. There was a period when radioactive cobalamin was unavailable and the corresponding absorption tests, including the Schilling test, were impossible to perform. However, ^57^Co-labeled vitamin can now be purchased (supplier MP Biomedicals, 29525 Fountain Parkway, Solon, OH 44139, USA [29].

The absorption of vitamin B_12 _from the intestine is reflected by a rise in serum levels of the vitamin bound to transcobalamin, 'holo-TC' following oral administration of the vitamin. First, a standard dose of non-radioactive cobalamin is given alone. If a sufficient increase in holo-TC level is not obtained, the test should be repeated, administering the vitamin bound to intrinsic factor. In intrinsic factor deficiency, holo-TC will rise. In malabsorption this rise is not observed. This relatively new test has been used successfully in the diagnosis of IGS [30].

A complicating factor is that enterocyte function is affected by cobalamin deficiency, causing secondary malabsorption. In this case, absorption tests may indicate general malabsorption and administration of intrinsic factor may fail to increase the absorption of radioactive cobalamin. Such tests should therefore be performed when the deficiency state has been corrected [2,31,32]. However, postponing or repeating the tests is time-consuming, collection of urine cumbersome and the costs may be high. The radiolabeled vitamin B_12 _absorption tests and intrinsic factor assays are often not available. Recently, the greatest problem has been to differentiate between IGS and intrinsic factor-deficiency [20]. Molecular analysis of the *CUBN*, *AMN *and *GIF *genes may therefore be the diagnostic method of choice [20]. Due to the uncertainty regarding the diagnosis, many reported cases need to be re-evaluated and data on the frequency of symptoms, such as proteinuria, may have to be corrected.

When found, proteinuria is strongly suggestive of IGS. However, the incidence seems to be lower than generally assumed. In a study on 13 Finnish cubilin-deficient patients, we found that moderate or clearly evident proteinuria was present in six of the subjects. In the patients with proteinuria, the excretion of total protein and albumin, and to a lesser extent transferrin, immunoglobulin light chains, and α_1_- and β_2_-microglobulins was increased. The urinary excretion of the cobalamin-intrinsic factor receptor was low in five of the patients studied. Light and electron microscopy of kidney biopsies revealed no or slight and uncharacteristic changes. These minor alterations were probably due to cobalamin deficiency rather than to true kidney disease [6]. Similar findings were made in a Norwegian study [7].

The cobalamin-intrinsic factor receptor is excreted in the urine and was found to be decreased in the cubilin-deficient Finnish patients [6,33]. Whether this test is useful in diagnosing patients with the *AMN *mutation is unknown.

When there is reasonable evidence to suspect that the patient suffers from IGS, a new and straightforward way to diagnosis is mutational analysis of the appropriate genes. If this molecular approach is unavailable, it is necessary to resort to the series of radiovitamin B_12 _and other tests described above, among which it would be advisable to include the assay of intrinsic factor in the gastric juice. However, as the treatment for both congenital lack of intrinsic factor and IGF is the same, there is no risk for the patient if one cannot distinguish between these two conditions.

## Treatment

As in genuine pernicious anemia, life-long treatment with vitamin B_12 _is necessary for IGS. The vitamin B_12 _deficiency is first corrected by giving intramuscular injections of cobalamin (1 mg of hydroxocobalamin daily for 10 days). It is recommended that these injections are then repeated once a month for the rest of the patient's life.

There are differences in opinion as to whether cyanocobalamin should be used to treat cobalamin deficiency. Cyanocobalamin is usually administered first as injections and then possibly orally in large doses. If injected, cyanocobalamin is poorly retained and some patients may fail to respond or even have side effects such as muscular pains, possibly due to inability to process cyanide. There is thus a case for withdrawal of this drug [34]. Oral treatment is based on the finding that when large doses of vitamin B_12 _are given orally, sufficient amounts are absorbed, even in the absence of intrinsic factor. Successful treatment of IGS with 1 mg of vitamin B_12_, orally administered at 2-week intervals, has been reported [30]. However, in early studies on Finnish patients, the absorption of milligram doses of dilute radioactive cobalamin was lower than that in pernicious anemia patients [31,32]. In view of the accumulating evidence that subclinical deficiency of cobalamin may contribute to the development of atherosclerosis, dementia and osteoporosis [3-5] and considering that cobalamin is non-toxic, it is suggested that patients receive a higher dose of cobalamin than necessary, rather than an insufficient dose.

The condition is rare, the first symptoms are vague and in theory, the deficiency may cause serious damage, especially to the brain. Early diagnosis is therefore important.

## Genetic counseling

The disease is transmitted as an autosomal recessive trait. Therefore, the relatives of the patients should be told about the disease and advised to mention the possibility of the disease to their physician.

## Prognosis

When sufficient amounts of vitamin B_12 _are supplied, the prognosis is excellent. The first cases have been observed for almost 50 years. When the deficiency state has been corrected, the only nutrient to be malabsorbed is vitamin B_12_; the proteinuria also persists. The proteinuria does not increase with time and kidney function does not deteriorate. The patient (or the parents) should be warned not to stop the treatment even though symptoms seem not to reappear immediately following cessation of therapy.

## Unresolved questions

The putative additional genes causing IGS should be identified and the safety of oral treatment needs to be better documented. Hopefully, the genetic tests will become easily available. The structures and modes of action of the intestinal cobalamin-intrinsic factor receptor and the tubular protein-receptor need to be elucidated.
